# Analysis of Gene Expression in an Inbred Line of Soft-Shell Clams (*Mya arenaria*) Displaying Growth Heterosis: Regulation of Structural Genes and the NOD2 Pathway

**DOI:** 10.1155/2016/6720947

**Published:** 2016-10-16

**Authors:** John J. Wilson, Janelle Grendler, Azaline Dunlap-Smith, Brian F. Beal, Shallee T. Page

**Affiliations:** ^1^Division of Environmental and Biological Sciences, University of Maine at Machias, Machias, ME 04654, USA; ^2^The Jackson Laboratory, Bar Harbor, ME 04609, USA; ^3^Mount Desert Island Biological Laboratory, Salisbury Cove, ME 04672, USA

## Abstract

*Mya arenaria* is a bivalve mollusk of commercial and economic importance, currently impacted by ocean warming, acidification, and invasive species. In order to inform studies on the growth of* M. arenaria*, we selected and inbred a population of soft-shell clams for a fast-growth phenotype. This population displayed significantly faster growth (*p* < 0.0001), as measured by 35.4% greater shell size. To assess the biological basis of this growth heterosis, we characterized the complete transcriptomes of six individuals and identified differentially expressed genes by RNAseq. Pathways differentially expressed included structural gene pathways. Also differentially expressed was the nucleotide-binding oligomerization domain 2 (NOD2) receptor pathway that contributes to determination of growth, immunity, apoptosis, and proliferation. NOD2 pathway members that were upregulated included a subset of isoforms of* RIPK2 *(mean 3.3-fold increase in expression),* ERK/MAPK14* (3.8-fold),* JNK/MAPK8* (4.1-fold), and* NFκB* (4.08-fold). These transcriptomes will be useful resources for both the aquaculture community and researchers with an interest in mollusks and growth heterosis.

## 1. Introduction

The soft-shell clam,* Mya arenaria, *is an infaunal, benthic marine bivalve that inhabits soft-sediments in the intertidal and shallow subtidal zone and ranges in the northwest Atlantic from North Carolina to Labrador [1–3].* M. arenaria* is a member of the phylum Mollusca, ranging throughout the northern, boreal coastline spanning several continents [1]. The clam is a filter-feeding bivalve, causing it to bioaccumulate environmental pollutants, and thus acts as a sentinel species [4]. In Maine,* M. arenaria *is harvested commercially, and, in 2015, the soft-shell clam fishery was the second most important in dockside value ($22.5 million) behind the American lobster,* Homarus americanus* ($495.4 million) [5]. Historically one of the most abundantly fished organisms caught along the coast of North America (over 1,200 metric tons in 2014 [6]),* M. arenaria* is prone to overharvest [7]. In spite of their importance, clams and the entire phylum Mollusca are understudied and underrepresented in GenBank [8].

At present, the basis for differential growth rates (growth heterosis) in mollusks is poorly understood [9], but differential production of specific proteins has been shown to modulate growth. For example, it has been shown that overexpression of salmon growth hormone is sufficient to increase growth in rainbow trout [10] and that in cell culture of* Pecten maximus L.* digestive gland cells, insulin and IGF-I, but not EGF and bFGF, stimulated proliferation [11]. In vertebrates, there are a number of genes, such as insulin-like growth factors (IGF) and IGF binding proteins, that have been found to be differentially expressed [12, 13]. Peterson et al. studied the differential mRNA expression of IGF-I and IGF-II in slow and fast-growth catfish [14]. Studies examining the differential gene expression in a FG phenotype in Pacific oyster* Crassostrea gigas* [15, 16] identified genes overrepresented in screens of growth heterosis, 50% of which were ribosomal proteins, indicating the importance of translational regulation in fast growth [17]. Several of these studies were on single growth factors, but Pace et al. [18] showed that a range of environmental and metabolic variables can affect growth heterosis and that these interactions are very complex. Other genes identified by Meyer and Manahan in fast-growth oysters included ATP synthase gamma, caveolin, and histone H2A [15]. Thus, based on these previous studies in other organisms, we hypothesized that specific growth-related genes would be differentially expressed in the FG clams, such as those coding for growth factors and ribosomal proteins [9–12, 14, 15].

To that end, we developed a fast-growth inbred line of clams (*M. arenaria*) by classical selection. Siphon tissue was chosen because siphon growth is a reliable proxy for total growth [19] and because siphon can be easily dissected as clean, homogenous tissue. Differential gene expression analysis (RNAseq) of the selected F3 generation compared to unselected F1 clams was conducted following* de novo* transcriptome assembly. Real-time quantitative reverse-transcription polymerase chain reaction (qPCR) was used for comparison. The genes related to growth were examined in both a fast-growth (FG) inbred F3 line and an unselected (F1) line of* M. arenaria*. Pathways overrepresented in the screen were analyzed in depth.

Understanding the expression patterns of key genes will help illuminate the mechanisms involved in these processes in bivalves and have important implications for maintenance of this important food-stock. Furthermore, studies of the molecular events associated with the growth process have important implications for the balance between apoptosis and cell proliferation in growth and cancer.

## 2. Materials and Methods

### 2.1. Animals

*M. arenaria* adults were produced at the Downeast Institute for Applied Marine Research and Education (DEI) (Beals, Maine, USA), our shellfish production and research center, at The University of Maine at Machias, where this species is routinely cultured for stock enhancement programs in coastal communities. Large individuals were hand-selected and inbred for two generations, as described below. All FG clams in this study were from the F3 generation. To avoid batch effects, FG and F1 were subjected to identical field conditions and assigned random numbers upon harvest. The double blinding was maintained until grouping for data analysis.

Beginning in 2002 with wild stocks taken from eastern Maine, adults were spawned, and their larvae and juveniles reared at DEI. Juveniles (F1 generation) were placed in a field-based nursery through the summer and fall, and then overwintered seed was planted in April 2003 in protected field plots at an intertidal site in the town of Beals, Maine, USA.

### 2.2. Selection, Growth, and Survival

The initial size of the clams was measured at the “hatchery mark,” an area of pitted and gouged shell that forms a band near the umbo when hatchery raised clams are seeded into the wild. This mark has been shown to accurately reflect the size of the clam at seeding [20].

In June 2005, approximately 300 F1 animals were removed from the plots, and the 30 largest clams (size range = 50–55 mm shell length (SL)) were selected and stimulated to spawn. The juveniles from that spawning (F2 generation) were reared similarly and seeded in protected field plots at an intertidal site in the town of Cutler, Maine, in April 2006. In June 2008, approximately 300 animals were removed from the field plots in Cutler and another selection was made of the 30 largest clams (size range = 52–58 mm shell length). Those animals were stimulated to spawn, producing an F3 generation. The F3 larvae and juveniles were treated similarly through the summer and fall and then overwintered. Also, in June 2008, wild clams collected from a clam buying station in the town of Beals were stimulated to spawn, and these F1 individuals were treated identically through the summer, fall, and winter. On 29 May 2009, a field experiment was conducted at Duck Brook Flat in the town of Cutler, Maine, USA, near the low water mark to determine if growth and/or survival of the F3 stock were different than the F1 stock. Clams (10–12 mm SL) from the FG and F1 lines were added at a density of 1,320 m^−2^ separately to plastic horticultural pots (experimental units) filled with ambient sediments and pushed into the sediments to within 5 mm of the rim. One-half of the experimental units were covered with a protective flexible plastic netting (6.4 mm), while the other half had no covering of netting. This factorial combination of treatments was replicated 10 times, and the forty experimental units were arrayed in a single 8 × 5 matrix with 1 m spacing between rows and columns. After 201 days, the experiment was concluded (15 December 2009) when all units were removed from the flat, and the contents of each washed through a 2 mm sieve. All live and dead animals were counted. The initial and final SL were measured to the nearest 0.1 mm for each live clam, and the wet mass of each live individual was recorded to the nearest 0.001 g. All FG clams in this study are F3 generation. F1 clams in this study were reared in the hatchery under identical conditions but not hand-selected for size.

### 2.3. Clam Dissection

Clams from DEI were transported and stored at 4–10 degrees Celsius in plastic bags with moist paper towels in the bottom to prevent desiccation. Clams (*n* = 6) were placed in a container and were immersed in 30 g/L MgCl_2_ in filter sterilized seawater for 5 minutes to anaesthetize the animals [21]. Clams were then sprayed with 95% ethanol. The siphon sheath was removed from the siphon and the siphon was rinsed in the seawater and then in RNAse-free water. A cross section of the siphon tip, containing the fused incurrent and excurrent siphons, was removed and placed in a 2 mL tube, filled with RNAlater to preserve RNA integrity, and was stored at −20°C for less than two months.

### 2.4. Genome Sequences

Genomic sequence for* M. arenaria* was generated and kindly provided by Dr. Charles W. Walker of the University of New Hampshire and the Hubbard Center for Genome Studies at the University of New Hampshire. We loaded a local instance of NCBI BLAST with the contigs of the* Mya arenaria* genome to match transcriptomic contigs with their corresponding genomic contigs. tBLASTx was used to identify annotated genes on NCBI that most closely matched the contigs. CLCBio NGS Genomics Workbench (QIAgen, Hilden, Germany) was used to align the genomic and the transcriptomic contigs so as to identify intron and exon regions of the genes of interest when needed.

### 2.5. RNA Prep for RNAseq

Inner siphon tips were dissected from six individual clams, three F1 and three F3 FG clams. Tissue was homogenized in the TissueLyser LT (QIAgen, Hilden, Germany) and was run at 50 Hz for six minutes. RNA was purified with TRIzol using RNeasy Fibrous Tissue Mini Kit (QIAgen). RNA was assessed for quality by Bioanalyzer (Agilent, Santa Clara, CA, USA) at Mount Desert Island Biological Laboratory (Salisbury Cove, ME, USA). Illumina TruSeq RNA sequencing stranded library construction and transcriptome sequencing were conducted at the Delaware Biotechnology Institute at the University of Delaware using an Illumina HiSeq2000 according to manufacturer's specifications.

### 2.6. RNAseq Analysis

FastQC was utilized to assess quality scores of RNAseq reads. Quality scores indicated no trimming to be necessary*. De novo* partial assembly was done using CLCBio. CLCBio NGS Genomics Workbench (v.6.0) (QIAgen) was used to compare transcript abundance between F1 and F3 transcriptomes. Expression value normalization is based on the reads per kilobase per million mapped reads (RPKM) to compensate for read length [22, 23]. The variance in transcripts between the FG (F3) selected transcriptome and the F1 transcriptome was analyzed using normalized Baggerley's test. A Bonferroni correction for multiple comparisons was applied subsequent to Baggerley's test for a more stringent screen. The cutoff point was Baggerley's/Bonferroni *p* less than or equal to 10^−7^.

Differentially expressed transcripts were categorized as matching annotated genes, as genes that are uncharacterized, or as having no matches. Uncharacterized genes were contigs that matched published genes in GenBank whose identity and function are not known. Placements were based on open tBLASTx searches of the GenBank database with an *E*-value threshold of *E* = 10^−4^. After identifying a pathway that was differentially expressed, the expression level of other members of the pathway was examined by creating a local BLAST database of all Mya contigs and searching for orthologues (mollusk sequences when possible, but more often other invertebrate or mammalian sequences were used when no molluscan orthologues were found in GenBank). For this* post hoc* screen, Baggerley's/Bonferroni *p* < 0.05 was used.

The volcano plot was generated using Bioconductor [24] (v.3.2). The RNAseq Illumina reads from the current project have been submitted to the NCBI SRA (Sequence Read Archive), BioProject accession number: PRJNA221373.

### 2.7. RNA Preparation for qPCR

A sample of siphon tip tissue of about 30 mg was placed in a 2 mL microcentrifuge tube along with a 5 mm stainless steel bead and 300 *μ*L of TRIzol reagent. The tubes were placed in the TissueLyser LT (QIAgen) and were run at 50 Hz for six minutes. The RNA was extracted using QIAgen RNAeasy Fibrous Tissue kit. 10 *μ*L DNase (Ambion, Life Technologies, Carlsbad, CA, USA) stock solution was added to prevent genomic DNA contamination. Quality was assessed by QIAExcel (QIAgen) and/or by agarose gel. Purified RNA was assayed by Nanodrop (Thermo Scientific, Waltham, MA, USA) and was frozen at −80°C, typically with RNasin RNAse (Promega Life Sciences, Madison, WI, USA) inhibitor.

### 2.8. cDNA Preparation

Two-step qPCR was performed for each sample: reverse-transcription followed by qPCR. A sample of the RNA was diluted to 100 ng/*μ*L and 1 *μ*L was then used in the cDNA reaction. The reaction was conducted with a ProtoScript II first strand cDNA synthesis kit (NEB, Ipswitch, MA, USA) that was used according to the manufacturer's specifications.

### 2.9. qPCR Reference Gene Validation and qPCR Conditions

The protocol and settings for the reference genes were optimized for temperature, primer concentration, and cDNA concentration and these genes were then run using cDNA from F1 clams and FG inbred line clams. Efficiency and linearity of linear fit for the cDNA concentration standard curve were assessed using standard techniques suggested by Bio-Rad (Hercules, CA, USA) [25]. Efficiency was calculated as Efficiency = −1 + 10^(−1/slope)^ and linearity of fit was assessed as *R* ^2^.

A subset of housekeeping genes from Araya et al. [26] was assessed for suitability as reference genes: actin gamma, elongation factor 1, ribosomal protein s-18 (RPS), and ubiquitin. PCR quantitation for these genes utilized primer sequences from Araya et al. [26] and they were synthesized by Integrated DNA Technologies (Coralville, Iowa). Genes were assessed for use as reference genes, along with candidate genes for differential expression.

Primers for experimental genes such as* BIRC2* were designed using NCBI primer BLAST, under default settings, with the assembled transcriptome used as a PCR template, and were further BLASTed against a genomic assembly for* Mya arenaria *to test for primer specificity in our organism of interest, although the lack of annotated genome does not allow us to exclude the possibility of pseudogenes or to design exon-spanning primers to limit amplification of some genomic DNA. Primers were synthesized by Integrated DNA Technologies (Coralville, Iowa, USA).

A series of controls were run to ensure qPCR optimization and accuracy. Every run included at least one well containing a no template control (NTC) to control for primer dimer formation and gDNA contamination. Agarose gels and melting curves were run with the products of each gene of interest to ensure that the primers were amplifying a single product. RNA purity and integrity was assessed via Thermo Scientific NanoDrop 2000c spectrophotometer and/or QiAxcel Advanced, using manufacturer specifications. Standard concentrations of primers were kept through all of the experiments, and template concentration was assessed Via NanoDrop and then standardized. qPCR was conducted using SYBR green master mix (Promega). C_*T*_ values, mean, standard deviation, and melting curves were generated by the instrument software CFX Manager (Bio-Rad). The C_*T*_ values between technical replicates were found to be consistent by coefficient of determination (*R* ^2^) and efficiency. Reference genes, whose expression levels were constant between F3 (FG) and F1 individuals, were used to normalize the data. For some runs, alien RNA from Alien QRT-PCR Inhibitor Alert (Agilent Technologies, Santa Clara, CA) was used as per instructions to control for inhibitors in the* M. arenaria* cDNA. All qPCR runs were conducted on a Bio-Rad MiniOpticon; the reference dye HEX with a sample run was used to control for laser variation of the MiniOpticon. The alien RNA, the RPS standard, the no reverse transcriptase control (NRC), and no template controls (NTC) using the same primer set were analyzed together in the same 48-deep well plate (Bio-Rad) in order to minimize run-to-run variations. The threshold level generated by the instrument curves was manually evaluated for each run and adjusted to meet the linear portion of the curve for determination of the threshold cycle values (C_*T*_). Parallel samples were processed using the same batch of reagents to minimize sample-to-sample variations.

### 2.10. Annotation and Gene Ontology

Gene annotation was carried out using the BLAST2GO program [27], FASTA-formatted sequences representing the unique upregulated transcripts were uploaded to the program, and BLASTX or BLASTn searches were carried out. Some data mining was performed using BLASTX through a CLCBio workflow (QIAgen). Gene Ontology for candidate genes was assessed using AmiGo at Gene Ontology (http://www.geneontology.org/), GoTermFinder [28], UniProt (http://www.uniprot.org/), and GO Slim (http://go.princeton.edu/cgi-bin/GOTermMapper) [29]. The NOD2-pathway figure was drawn using Pathvisio (http://www.pathvisio.org/).

### 2.11. STRING

The network analysis was conducted with the software STRING v.10 [30]. We chose the top 100 most BLAST hits with the greatest expression difference. We searched for network interactions using the closest annotated genome in STRING, the purple sea urchin,* Strongylocentrotus purpuratus* (BioProjects #* PRJNA13728*,* PRJNA56067*, and* PRJNA10736*). We filtered the STRING v.8 human interactome to include only interactions which had a confidence score ≥0.4 (medium stringency).

## 3. Results

### 3.1. Growth Heterosis

**Figure 1:**
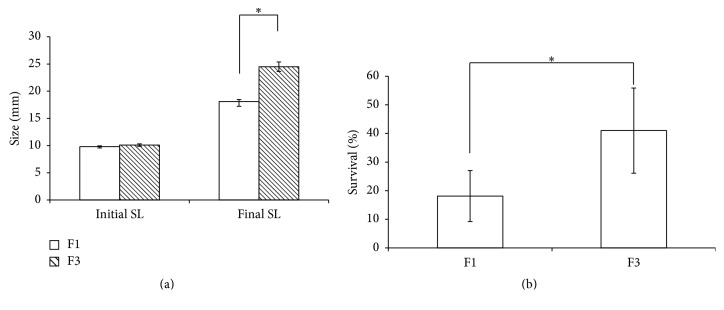
Growth and survival of inbred line of* M. arenaria*. (a) Size, measured in shell length (SL) (in mm, open bars) and growth, measured as mean final SL (in mm, diagonally hatched bars) in the F3 versus F1* Mya arenaria*. Error bars represent 95% CI (^*∗*^ *p* < 0.0001). Growth was 35.4% greater in the F3 versus F1 line. (b) Mean percent survival (open bars) in F3 versus F1* Mya arenaria*. Mean percent survival was greater in the F3 versus F1 line by 126.4% (^*∗*^ *p* = 0.011).

We examined the possibility of enhancing shell growth in* M. arenaria* through classic selective breeding. Selection resulted in a clam inbred line that displayed growth heterosis as assessed by shell length (SL) (Figure 1).

Growth, measured as mean final SL, was 35.4% greater in the F3 versus F1 line (18.1 ± 0.38 versus 24.5 ± 0.88) (Figure 1(a)). Also, mean percent survival was greater in the F3 versus F1 line by 126.4% (18 ± 8.9 versus 41 ± 14.9) (Figure 1(b)). Survival was not enhanced by netting (*p* = 0.3968) due to the accidental inclusion of green crab juveniles in seven of the ten netted units for both F1 and F3 line treatments. No differences were observed between selection lines in the mass-length relationship suggesting that selection for increased rate of shell production did not negatively interfere with tissue mass or growth. Notably, when released from artificial selection, in less than two generations, the FG inbred line was no longer significantly larger than the matched F1 population (dns).

### 3.2. RNAseq

Complete transcriptomes were sequenced from three F1 individuals and three FG individuals. The Phred quality scores from the paired-end reads were above 30 to 150 bp, indicating a base call accuracy of at least 99.9% (Figure S1, in Supplementary Material available online at http://dx.doi.org/10.1155/2016/6720947). In the absence of a reference genome,* de novo* assembly was performed. A total of 122,012,641 matched, paired-end reads were available for contig assembly and mapping, with a median length of 142 bp (Figure S2). Assembly utilized a cutoff of ≥200 bp to minimize low information assemblies. The assembly resulted in an average of 79,470 contigs with a maximum size of 25,395 bp and N50 of 1037 bp.

We identified 415 differentially expressed genes between the F1 and FG clams (Bonferroni *p* value < 0.05). A volcano plot showed that the most highly differentially expressed genes were downregulated (Figure S3). The genes were sorted by Bonferroni *p* value and then sorted by fold-expression difference between F1 and FG inbred lines. More than 50% of the contigs had no useable BLAST hits (BLAST cutoff *E* = 10^−10^) due to noncoding transcripts, transcriptome contigs that only represented untranslated regions, and poor sequence database representation of mollusks. Of the 415 unique, significantly differentially expressed transcripts in FG clams, putative annotation could be determined for 162 based on sequence similarity by BLASTX searches while the majority had no significant similarity to protein sequences in the nr database (cutoff *E*-value = 10^−10^). Genes with the largest positive and negative expression differences were often structural genes (Table 1).

### 3.3. Real-Time Quantitative PCR

To validate the differential expression observed by RNAseq, we chose an upregulated gene from our RNAseq data: baculoviral IAP repeat-containing protein 2 (*BIRC2*), (also known as* c-IAP* or* IAPOP1*).* BIRC2 *was of interest due to its position in the upregulated NOD2 pathway, as well as its physiological role in growth, immunity, and apoptosis. Comparing FG F3 individuals with F1 individuals,* BIRC-2* demonstrated a ΔΔC_*T*_ (±SD) = 7.6 (±2.02). This result was consistent with the differential expression seen in our RNAseq, but the fold-difference was smaller when assayed by qPCR. The ribosomal protein* S3A* (*RPS*) was chosen as a reference gene, based on C_*T*_ below 25, constant expression between individuals and between experimental and control groups, consistent melt temperature, and amplification efficiency (Table S1A). Between individuals, both FG and WT, the fold change was 0.3 ± 1.4 (Table S1B).

### 3.4. Gene Ontology and Network Analysis

**Figure 2:**
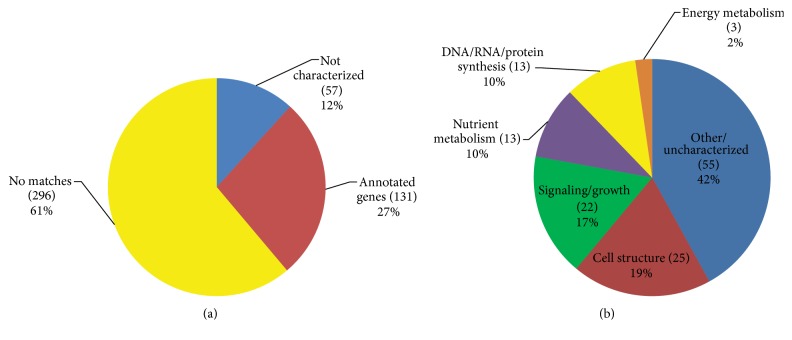
Categorization of differentially expressed genes from FG* Mya arenaria.* (a) Summary of identification of top 484 differentially expressed genes. (b) Summary of categories of Gene Ontology (GO) for the genes from (a) that were successfully annotated. List of 131 different genes identified were annotated with Gene Ontology biological process terms.

In order to categorize the function of the genes in our differential expression of RNAseq screen, we took 484 genes with the highest differences in expression between F3 FG and F1 clams. Of those genes with useable BLAST hits, 19% had Gene Ontology related to cell structure, 17% related to signaling or growth, 10% related to nutrient metabolism, 10% related to synthesis of critical macromolecules, and 2% related to energy metabolism (Figure 2).

**Figure 3:**
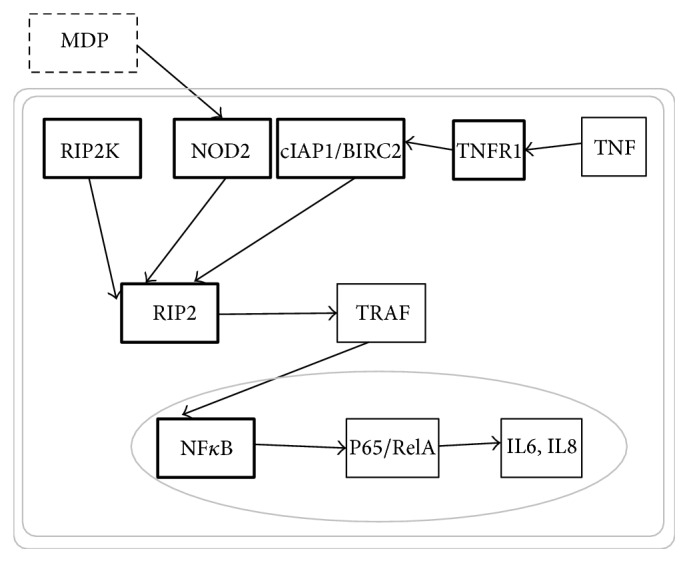
Significantly differentially regulated genes in* NOD2* Pathway. Genes significantly upregulated in the RNAseq screen (heavily outlined boxes).* Post hoc* analysis found notable differences in expression level in* RIPK2* nine isoforms (five with average 3.3-fold increase, average *p* value 0.053, three isoforms ns, and one with 1.5-fold change),* ERK/MAPK14* isoforms (one with 3.8-fold increase, *p* < 0.002, three ns),* JNK/MAPK8* three isoforms (fold difference 4.1, *p* < 0.009, two other isoforms ns), and* NFκB* (fold difference 4.08, *p* < 0.04). The following showed no significant difference in expression level:* ERBIN*,* iKKB*,* CASP8, TAK1*,* TRAF6*,* TRIP6*,* SGT1*, and* CARD6*.

Metabolic genes represented 21% of the characterized genes. For example, fatty acid synthase transcripts were 22-fold higher in the FG individuals (*p* < 10^−274^). By manual inspection, we also determined that five of the transcripts for the NOD-like receptor signaling pathway were differentially expressed in the screen (Figure 3).

A* post hoc *search of our RNAseq results for other pathway members yielded three that were consistently and significantly upregulated (*NFκB*,* JNK*, and* ERK*), one that had multiple isoforms upregulated but none of which reached significance singly (*RIPK2*), and eight other related proteins that were not significantly differentially expressed (*ERBIN*,* iKKB*,* CASP8*,* TAK1*,* TRAF6*,* TRIP6*,* SGT1*, and* CARD6*). One intermediate member of the pathway was not represented: no BLAST hits corresponded to* TRAF*.

**Figure 4:**
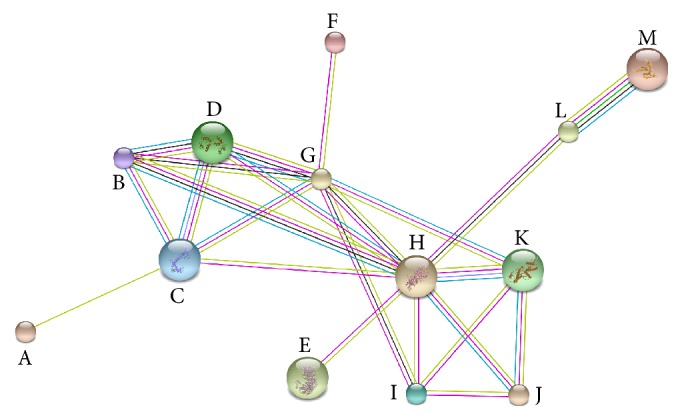
Gene network analysis using STRING. A red line indicates the presence of fusion evidence; a green line indicates neighborhood evidence; a blue line indicates cooccurrence evidence; a purple line indicates experimental evidence; a yellow line indicates text-mining evidence; a light blue line indicates database evidence; a black line indicates coexpression evidence. Gene identification: A: Farnesyl diphosphate farnesyltransferase; B: Twitchin-like; C: Calmodulin; D: Myosin light chain; E: K14280 Exportin-like; F: PolyBC binding protein-like; G: Myosin heavy chain; H: Actin-related protein 1; I: Microtubule-associated monooxygenase- calponin- and LIM-domain-containing protein; J: Actin-related protein 2/3 (*Arp 2/3*); K: Actin-related protein 2a-like; L: Elongation factor-2-like; M: 40S ribosomal protein S2-like.

The cluster analysis in STRING draws association data from several databases, including the Kyoto Encyclopedia of Genes and Genomes (KEGG) [30], and was used to establish genes sharing a common biological pathway (Figure 4).

The most complete network of differentially expressed genes was involved in cytoskeletal processes and protein translation.

## 4. Discussion

We demonstrated that the F3 generation of classical selection was sufficient to generate transient growth heterosis. To the best of our knowledge, this is the first-ever demonstration of artificial selection for any morphological attribute in* Mya arenaria*. Coastal communities incorporating stock enhancement with cultured soft-shell clam juveniles may enjoy greater production in areas seeded with animals that have been genetically selected for fast growth.

Because we were interested in the physiological origin of the growth heterosis, we chose to assay for differential gene expression. Over just three generations, we did not expect heritable changes. In addition, once the selection was released, the clam size reverted to the mean in F4. This suggests that we have been selecting for higher gene expression.

The preponderance of the differentially expressed genes in the FG clams was structural genes. Curiously, in the FG clams, these genes are strongly and consistently downregulated. For example, myosins, actins, microtubules, and several related genes appear in the screen downregulated. One might expect that production of a number of the building blocks for growth of the organism would be increased to meet demand of growth—or, at least, maintained as a housekeeping function. However, there are numerous studies where strong variation in actin gene expression has been seen (e.g., [31–33]). In our hands, the RT-qPCR actin in the soft-shell clams proved to have significant individual variation and was rejected as a normalization gene (Table S1A). Furthermore, in growth states—particularly cancer—there are numerous examples of structural genes differentially regulated [34], although upregulation is perhaps more common, particularly with wounding or remodeling [35–37].

About a quarter of the characterized differentially expressed genes were metabolic genes. For example, two isoforms of fatty acid synthase, ATPase and elongation factor 2 (*EF2*), are all represented in the most significantly differentially expressed genes. This is consistent with other studies on growth heterosis that emphasize the importance of protein synthesis genes and protein processing [18] and turnover [17, 38].

The genes in the NOD-like receptor signaling pathway were overrepresented in the differential expression screen. The NOD pathway forms an interesting crossroads between innate immunity, growth, and apoptosis. Unfortunately, we were unable to find the sequence of some critical* NOD2* players in the transcriptome, even though some have been found previously in scallops [39].* RIPK2* is an interesting gene in this analysis because it is a convergence point for upstream genes that are differentially expressed. The screen pulled up seven contigs that mapped to* RIPK2* most of which were not significant but six of the seven showed upregulation and near significance. In addition to contributing to growth, the* NOD2* pathway leads to transcription of proinflammatory cytokines via TNF-alpha and* NFκB* [40]. The pathway has been implicated in the inflammatory bowel condition Crohn's Disease [41–43] and in cancers, particularly colorectal cancer [42, 44]. Other immune-related genes differentially expressed include a pathogenesis-related protein that was upregulated eightfold and interferon alpha-inducible protein that was downregulated 19-fold. The upregulation of immunity pathways is particularly interesting given the increased survival in the fast-growth F3 individuals seen in Figure 1(b). These results suggest that the regulatory genes in the NOD-like receptor signaling pathway may play a role in growth but we have no way of determining cause and effect from these data. Alternatively, it is possible that the upregulation of the innate immunity prevents pathogen invasion that would have otherwise limited growth; conversely, it is possible that the higher growth rate results in more pathogen exposure, which in turn upregulates the innate immune pathway.

We chose to validate BIRC2 by qPCR because it is an upregulated member of the NOD2 pathway at a crossroads between growth, immunity, and cancer, yet BIRC2 has been reported to have no phenotype when knocked down in* C. elegans *[45]. This lack of phenotype is in part because BIRC2 appears to be functionally redundant with BIRC1 in mouse knockouts, though their regulation depends on cell type [46].

The connection between growth pathways and cancer pathways is not unexpected. Interestingly, recent work has shown that* M. arenaria* is one of only three organisms shown to be susceptible to transmissible cancers. Metzger et al. [47] identified a line of clonal* M. arenaria* cells that are at least partially responsible for the high prevalence of hemocyte cancers in clams along the coast of the Northeast. It is possible in current siphon liquid or perfused hemocytes could contain high copy number of cancerous hemocytes. However, because we used washed siphon tissue for the transcriptome, we do not anticipate significant artifactual RNA from possible cancer cells.

As a nonmodel organism,* Mya arenaria* presents obstacles to analysis. Genomic data is limited and nucleotide divergence in mollusks is high [48]. The high number of unannotated or uncharacterized genes in the screen limits the scope of our interpretations, primarily due to noncoding genes and having some fragmented transcripts where the contigs only contain the UTR portion of the transcripts. The representation of mollusks in the NCBI database is low, particularly some of the bivalves of interest [49]. There may be overrepresented genes or pathways that are not well annotated. For this reason, network analysis must be interpreted conservatively.

To analyze organismal gene expression, we turned to high-throughput transcriptome analysis (RNAseq). Lack of representation in the database of annotated mollusks prevented us from identifying over half of the transcripts.

We determined that* RPS3A *served as a stable gene in the qPCR analysis and chose* BIRC2* as a differentially expressed gene to analyze. Between individuals, both FG and WT, the RPS showed low variability with a single melt peak and a single band by DNA analyzer.* RPS3A* produces a ribosomal protein that is a component of the small ribosomal subunit. It is a member of the S3AE family of ribosomal proteins and is located in the cytoplasm. In the realm of FG phenotype,* RPS3A* is an interesting gene. On one hand, it has appeared in screens for genes highly associated with growth heterosis [15] but* RPS3A* has also been used in screens of FG cells, especially cancer, as a housekeeping gene, as seen in a meta-analysis by Popovici et al. [50].

The upregulation of the* BIRC2* gene seen in both the RNAseq screen and the qPCR was of particular interest because the gene product BIRC2 lies at a crossroads between growth, cancer, and immunity. The sevenfold increase in* BIRC-2* expression assayed by qPCR was consistent with the differential expression seen in the RNAseq, but the fold-difference was smaller, a result that has been seen in other RNAseq/qPCR comparisons [51] and in part reflects the difference in the dynamic range of the two methods [22]. The* BIRC-2* knockout has been reported to have no phenotype [45, 52], so future interventions combining a knockdown with an immune challenge could prove instructive in evaluating the interactions between growth and innate immunity and in evaluating the partial functional redundancy of BIRC1 and BIRC2.

## 5. Conclusions

Our sequence database contributions and annotation will serve to improve the bivalve representation in GenBank (BioProject accession # PRJNA221373, SRA accession numbers # SAMN02361211-16). Our results show that suites of genes involved in structural remodeling, signaling, and apoptosis correlate with a fast-growth phenotype. Functional analysis of some of these genes, such as* BIRC-2,* will inform analysis of growth regulation in these ecologically and economically important species. Since these genes lie at the crossroads of immunity, growth, and cancer, there are a range of biomedical implications. In addition, elucidation of growth in bivalves can have implications for conservation and policy for* M. arenaria*.

## Supplementary Material

Supplemental material includes: RNAseq quality control data, PCR conditions and data, and a plot of global transcriptional changes seen in our RNAseq.

## Figures and Tables

**Table 1:** Most highly ranked differentially expressed genes in FG clams, listing Bonferroni *p* value and fold-difference compared to WT.

Gene name	*p* value	Fold change in expression
Gamma receptor epsilon-like	0	30.98
Fatty acid synthase-like-1	0	22.1
Fatty acid synthase-like-2	0	19.6
Cyclin Y-like	0	11.4
Vesicle glycoprotein	0	9.78
Pathogenesis-related	0	8.4
Aryl hydrocarbon receptor-like	0	8.3
RISC component-like	0	7.6
Leukocyte member	0	6.5
Na/K ATPase	0	6.3
Insulin-like GF receptor	0	6.1
cAMP response binding protein	0	5.7
NaCl/amino acid transporter	0	5.2
Actin-binding protein	0	5.1
Na/K ATPase	0	5.1
O-glucanase-like	0	4.6
Amyloid beta-like	0	4.4
Solute carrier family 13-like	0	4.3
Cathepsin-like	0	4.1
Rho GTPase	0	3.8
Elongation factor 2	0	3.7
Cold shock protein	0	3.4
Nuclear factor 1A	0	2.7
Tubulin alpha4a	0	2.6
Myosin heavy chain	0	−64.8
Otoferlin-like	5.*E* − 279	−42
Actin 2	1.*E* − 63	−290
Sarcoplasmic Calcium Binding Protein	4.*E* − 59	−5
Cytochrome P450	7.*E* − 49	−4
Actin	5.*E* − 38	−140
Interferon alpha-inducible protein 27	5.*E* − 36	−19
Exportin-1	2.*E* − 32	−21
Troponin I	1.*E* − 21	−6
Myosin heavy chain	1.*E* − 18	−9
Fatty acid synthase-like	2.*E* − 16	−45
Low density lipoprotein receptor	2.*E* − 16	−44
Elongation factor 2	2.*E* − 16	−3
Calmodulin	3.*E* − 16	−21
Alpha tubulin	2.*E* − 15	−83
Lysosome-associated membrane glycoprotein 1	5.*E* − 15	1
Fatty acid synthase	1.*E* − 14	−3
Transcriptional activator protein Pur-beta-like	2.*E* − 14	−8
Solute carrier family 13	5.*E* − 14	−34
Lysosomal-associated transmembrane protein 4A-like	6.*E* − 14	7
Bifunctional protein NCOAT-like	7.*E* − 14	−12
Far upstream element-binding protein 2-like	1.*E* − 13	19
Bifunctional protein NCOAT-like	3.*E* − 13	5
Patched domain-containing protein 3-like	4.*E* − 13	−10
Muscle blind-like protein 2-like	9.*E* − 13	−6
Beta-hexosaminidase-like	1.*E* − 12	−28
40S ribosomal protein S2	1.*E* − 12	7
Farnesyltransferase/geranylgeranyltransferase type-1 subunit alpha-like	6.*E* − 12	5
Saposin-related protein	7.*E* − 12	6
Chaoptin-like	7.*E* − 12	7
Tubulin, alpha 1	9.*E* − 12	−24

## References

[1] Snelgrove P. V. R., Grant J., Pilditch C. A. (1999). Habitat selection and adult-larvae interactions in settling larvae of soft-shell clam Mya arenaria. *Marine Ecology Progress Series*.

[2] Beal B. F., Parker M. R., Vencile K. W. (2001). Seasonal effects of intraspecific density and predator exclusion along a shore-level gradient on survival and growth of juveniles of the soft-shell clam, *Mya arenaria* L., in Maine, USA. *Journal of Experimental Marine Biology and Ecology*.

[3] Beal B. F., Gayle Kraus M. (2002). Interactive effects of initial size, stocking density, and type of predator deterrent netting on survival and growth of cultured juveniles of the soft-shell clam, *Mya arenaria* L., in eastern Maine. *Aquaculture*.

[4] Greco L., Pellerin J., Capri E. (2011). Physiological effects of temperature and a herbicide mixture on the soft-shell clam *Mya arenaria* (Mollusca, Bivalvia). *Environmental Toxicology and Chemistry*.

[5] Commericial Fishing Landings Data: Maine Department of Marine Resources, 2015, http://www.maine.gov/dmr/commercial-fishing/landings/index.html

[6] Annual Commercial Landing Statistics, National Marine Fisheries Service, NOAA Office of Science and Technology. https://www.st.nmfs.noaa.gov/commercial-fisheries/commercial-landings/annual-landings/index.

[7] Steneck R. S., Hughes T. P., Cinner J. E. (2011). Creation of a gilded trap by the high economic value of the maine lobster fishery. *Conservation Biology*.

[8] Bassaglia Y., Bekel T., Da Silva C. (2012). ESTs library from embryonic stages reveals tubulin and reflectin diversity in *Sepia officinalis* (Mollusca—Cephalopoda). *Gene*.

[9] Nijhout H. F., Davidowitz G., Roff D. A. (2006). A quantitative analysis of the mechanism that controls body size in Manduca sexta. *Journal of Biology*.

[10] Sekine S., Mizukami T., Nishi T. (1985). Cloning and expression of cDNA for salmon growth hormone in *Escherichia coli*. *Proceedings of the National Academy of Sciences of the United States of America*.

[11] Giard W., Lebel J.-M., Boucaud-Camou E., Favrel P. (1998). Effects of vertebrate growth factors on digestive gland cells from the mollusc *Pecten maximus* L.: an in vitro study. *Journal of Comparative Physiology B*.

[12] Bower N. I., Li X., Taylor R., Johnston I. A. (2008). Switching to fast growth: the insulin-like growth factor (IGF) system in skeletal muscle of Atlantic salmon. *Journal of Experimental Biology*.

[13] Peterson B. C., Waldbieser G. C., Bilodeau L. (2004). IGF-I and IGF-II mRNA expression in slow and fast growing families of USDA103 channel catfish (Ictalurus punctatus). *Comparative Biochemistry and Physiology A: Molecular and Integrative Physiology*.

[14] Peterson B. C., Waldbieser G. C., Bilodeau L. (2004). IGF-I and IGF-II mRNA expression in slow and fast growing families of USDA103 channel catfish (*Ictalurus punctatus*). *Comparative Biochemistry and Physiology Part A: Molecular & Integrative Physiology*.

[15] Meyer E., Manahan D. T. (2010). Gene expression profiling of genetically determined growth variation in bivalve larvae (*Crassostrea gigas*). *Journal of Experimental Biology*.

[16] Hedgecock D., Lin J.-Z., DeCola S. (2007). Transcriptomic analysis of growth heterosis in larval Pacific oysters (*Crassostrea gigas*). *Proceedings of the National Academy of Sciences of the United States of America*.

[17] Hedgecock D., McGoldrick D. J., Manahanb D. T., Vavrab J., Appelmansb N., Baynec B. L. (1996). Quantitative and molecular genetic analyses of heterosis in bivalve molluscs. *Journal of Experimental Marine Biology and Ecology*.

[18] Pace D. A., Marsh A. G., Leong P. K., Green A. J., Hedgecock D., Manahan D. T. (2006). Physiological bases of genetically determined variation in growth of marine invertebrate larvae: a study of growth heterosis in the bivalve *Crassostrea gigas*. *Journal of Experimental Marine Biology and Ecology*.

[19] Zwarts L., Wanink J. (1989). Siphon size and burying depth in deposit- and suspension-feeding benthic bivalves. *Marine Biology*.

[20] Beal B. F., Bayer R., Kraus M. G., Chapman S. R. (1999). A unique shell marker in juvenile, hatchery-reared individuals of the softshell clam, *Mya arenaria* L.. *Fishery Bulletin*.

[21] Heasman M. P., O'Connor W. A., Frazer A. W. J. (1995). Induction of anaesthesia in the commercial scallop, *Pecten fumatu*s Reeve. *Aquaculture*.

[22] Mortazavi A., Williams B. A., McCue K., Schaeffer L., Wold B. (2008). Mapping and quantifying mammalian transcriptomes by RNA-Seq. *Nature Methods*.

[23] Conesa A., Madrigal P., Tarazona S. (2016). A survey of best practices for RNA-seq data analysis. *Genome Biology*.

[24] Gentleman R. C., Carey V. J., Bates D. M. (2004). Bioconductor: open software development for computational biology and bioinformatics. *Genome Biology*.

[25] Larionov A., Krause A., Miller W. R. (2005). A standard curve based method for relative real time PCR data processing. *BMC Bioinformatics*.

[26] Araya M. T., Siah A., Mateo D. (2008). Selection and evaluation of housekeeping genes for haemocytes of soft-shell clams (*Mya arenaria*) challenged with *Vibrio splendidus*. *Journal of Invertebrate Pathology*.

[27] Conesa A., Götz S., García-Gómez J. M., Terol J., Talón M., Robles M. (2005). Blast2GO: a universal tool for annotation, visualization and analysis in functional genomics research. *Bioinformatics*.

[28] Boyle E. I., Weng S., Gollub J. (2004). GO::TermFinder—open source software for accessing gene ontology information and finding significantly enriched gene ontology terms associated with a list of genes. *Bioinformatics*.

[29] Gene Ontology Consortium (2004). The Gene Ontology (GO) database and informatics resource. *Nucleic Acids Research*.

[30] Jensen L. J., Kuhn M., Stark M. (2009). STRING 8—a global view on proteins and their functional interactions in 630 organisms. *Nucleic Acids Research*.

[31] Bustin S. A., Benes V., Garson J. A. (2009). The MIQE guidelines: minimum information for publication of quantitative real-time PCR experiments. *Clinical Chemistry*.

[32] Huggett J., Dheda K., Bustin S., Zumla A. (2005). Real-time RT-PCR normalisation; strategies and considerations. *Genes and Immunity*.

[33] Dheda K., Huggett J. F., Bustin S. A., Johnson M. A., Rook G., Zumla A. (2004). Validation of housekeeping genes for normalizing RNA expression in real-time PCR. *BioTechniques*.

[34] de Kok J. B., Roelofs R. W., Giesendorf B. A. (2005). Normalization of gene expression measurements in tumor tissues: comparison of 13 endogenous control genes. *Laboratory Investigation*.

[35] Deindl E., Boengler K., van Royen N., Schaper W. (2002). Differential expression of GAPDH and *β*-actin in growing collateral arteries. *Molecular and Cellular Biochemistry*.

[36] Toyofuku T., Hoffman J. R., Zak R., Carlson B. M. (1992). Expression of *α*-cardiac and *α*-skeletal actin mRNAs in relation to innervation in regenerating and non-regenerating rat skeletal muscles. *Developmental Dynamics*.

[37] Khan S. A., Tyagi M., Sharma A. K. (2014). Cell-type specificity of *β*-actin expression and its clinicopathological correlation in gastric adenocarcinoma. *World Journal of Gastroenterology*.

[38] Hawkins A. J. S., Day A. J. (1996). The metabolic basis of genetic differences in growth efficiency among marine animals. *Journal of Experimental Marine Biology and Ecology*.

[39] Wang M., Yang J., Zhou Z. (2011). A primitive Toll-like receptor signaling pathway in mollusk Zhikong scallop *Chlamys farreri*. *Developmental and Comparative Immunology*.

[40] Lawrence T. (2009). The nuclear factor NF-*κ*B pathway in inflammation. *Cold Spring Harbor Perspectives in Biology*.

[41] Xavier R. J., Podolsky D. K. (2007). Unravelling the pathogenesis of inflammatory bowel disease. *Nature*.

[42] Strober W., Murray P. J., Kitani A., Watanabe T. (2006). Signalling pathways and molecular interactions of NOD1 and NOD2. *Nature Reviews Immunology*.

[43] Pandey A. K., Yang Y., Jiang Z. (2009). Nod2, Rip2 and Irf5 play a critical role in the type I interferon response to *Mycobacterium tuberculosis*. *PLoS Pathogens*.

[44] Branquinho D., Freire P., Sofia C., De Gastroenterologia S. (2016). NOD2 mutations and colorectal cancer—where do we stand?. *World Journal of Gastrointestinal Surgery*.

[45] Fraser A. G., James C., Evan G. I., Hengartner M. O. (1999). Caenorhabditis elegans inhibitor of apoptosis protein (IAP) homologue BIR-1 plays a conserved role in cytokinesis. *Current Biology*.

[46] Giardino Torchia M. L., Conze D. B., Ashwell J. D. (2013). c-IAP1 and c-IAP2 redundancy differs between T and B cells. *PLoS ONE*.

[47] Metzger M. J., Reinisch C., Sherry J., Goff S. P. (2015). Horizontal transmission of clonal cancer cells causes leukemia in soft-shell clams. *Cell*.

[48] Hebert P. D. N., Cywinska A., Ball S. L., deWaard J. R. (2003). Biological identifications through DNA barcodes. *Proceedings of the Royal Society B: Biological Sciences*.

[49] Astorga M. P. (2014). Genetic considerations for mollusk production in aquaculture: current state of knowledge. *Frontiers in Genetics*.

[50] Popovici V., Goldstein D. R., Antonov J., Jaggi R., Delorenzi M., Wirapati P. (2009). Selecting control genes for RT-QPCR using public microarray data. *BMC Bioinformatics*.

[51] Palstra A. P., Beltran S., Burgerhout E. (2013). Deep RNA sequencing of the skeletal muscle transcriptome in swimming fish. *PLoS ONE*.

[52] Guicciardi M. E., Werneburg N. W., Bronk S. F. (2014). Cellular Inhibitor of Apoptosis (cIAP)-mediated ubiquitination of phosphofurin acidic cluster sorting protein 2 (PACS-2) negatively regulates tumor necrosis factor-related apoptosis-inducing ligand (TRAIL) cytotoxicity. *PLoS ONE*.

