# Supplementary material for: Analysis of Gene Expression in an Inbred Line of Soft-Shell Clams (Mya arenaria) Displaying Growth Heterosis: Regulation of Structural Genes and the NOD2 Pathway
Source: Int J Genomics. 2016 Oct 16;2016:6720947. doi: 10.1155/2016/6720947 (PMC5086354; doi:10.1155/2016/6720947)
Supplement: Supplementary file 1 — Supplemental material includes: RNAseq quality control data, PCR conditions and data, and a plot of global transcriptional changes seen in our RNAseq. [file 6720947.f1.pdf]

Supplementary Figure S1.

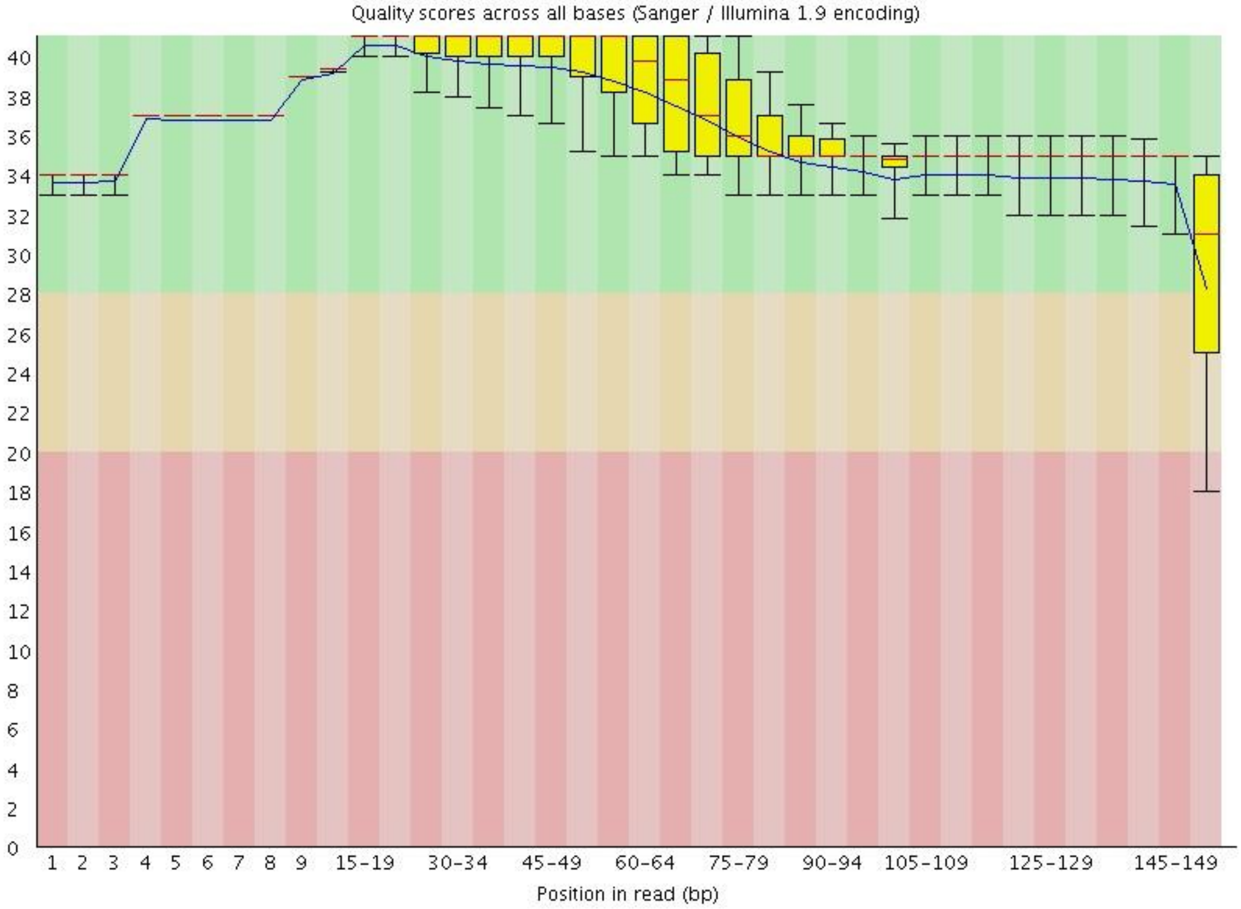

Supplemental Figure S1

RNA-seq base-quality distribution. The Phred read quality scores for the paired-end RNAseq were plotted in FastQC (R). Quality scores are plotted against the position in each read. Error bars = SEM. The background of the graph divides the y axis into very good quality (green), reasonable quality (orange), and poor quality (red).

Supplementary Figure S2

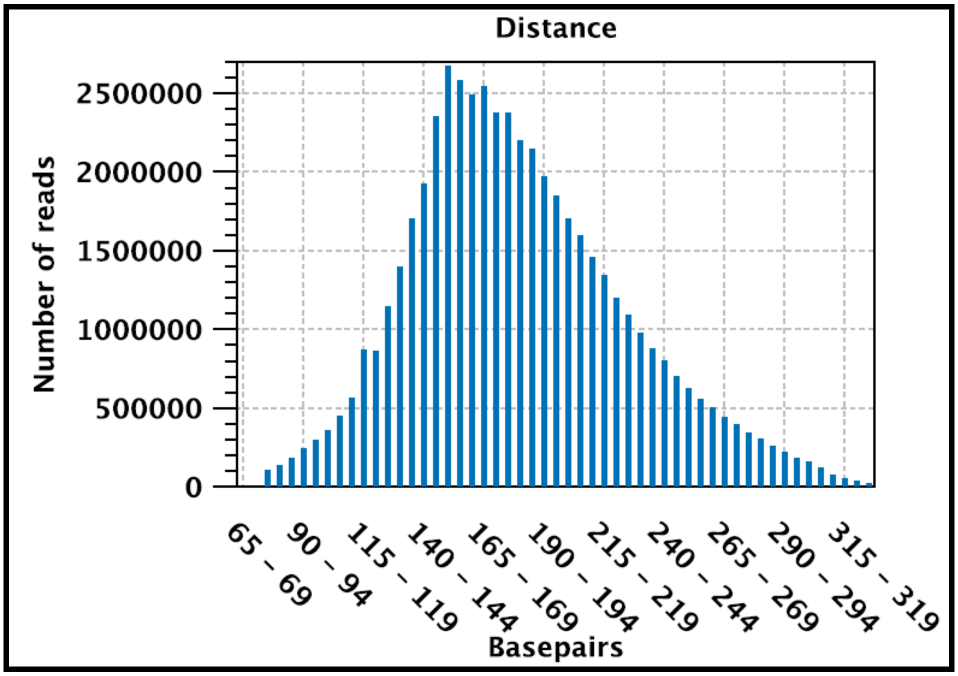

Supplementary Figure S2

Read distribution plot. The number of reads (abundance) was plotted versus the nucleotide position in the read. The plot was generated in CLCBio.

A.

Primer	Primer Sequence	Tm (°C)	Efficiency (%)	C_T
ActinF1	5’GCGAAAATACTCCGTCTGGA	60	N.D.	29.78-35.24
ActinR1	5’GCAGGTACGATCACAAGCAA	60		
EF1F1	5’GGTGGCTGTTGGTGTCATC	60	70.2	20.32-22.55
EF1R1	5’GGCCTAGGTGTTTTCCATGA	60		
UQF1	5’TCGCTAAGGAGCTGGACATT	60	214	32.40-39.99
UQR2	5’ACCGTCGCTCCTTGTACATC	60		
RPSF1	5’AAGATTCCCGACTGGTTCCT	60	92-97	19.19-24.96
RPSR1	5’GCCGGTTGTCTTTGTATGCT	60		

B.

Primer	Primer Sequence	Tm (°C)	Efficiency (%)	$\Delta\Delta C_T$ (+/-S.D.)
RPSF1	5’AAGATTCCCGACTGGTTCCT	51.8	92-97	0.3
RPSR1	5’GCCGGTTGTCTTTGTATGCT	51.8		+/- 1.4
BIRC2-F1	5’GATGGAAGACGACCCTGACC	60.3	103-118	7.60
BIRC2-R2	5’ACGTCCGCTTTCCTATCCAG	60.5		+/-2.02

Supplemental Table S1.

Quantitative PCR primer sequences and conditions

A. Primers from Araya et al. (2008) tested for suitability as reference genes in qPCR. Experiments were conducted as listed in “Methods” using SYBR green as a probe. Tm is the expected melting temperature reported by Araya et al (2008). Efficiency is efficiency of qPCR amplification reported as a percent. C_T is the threshold level of amplification reported as a range including individual variation. N.D. indicates “not determined”. Note that EF1 also produced duplicate melt curves.

B. The relative abundance of BIRC2 mRNA, compared to RPS mRNA abundance, was determined by qRT-PCR. Experiments were conducted as listed in “Methods” using SYBR green as a probe. Data is reported as mean ± standard deviation for three experimental replicates

Supplemental Figure S3

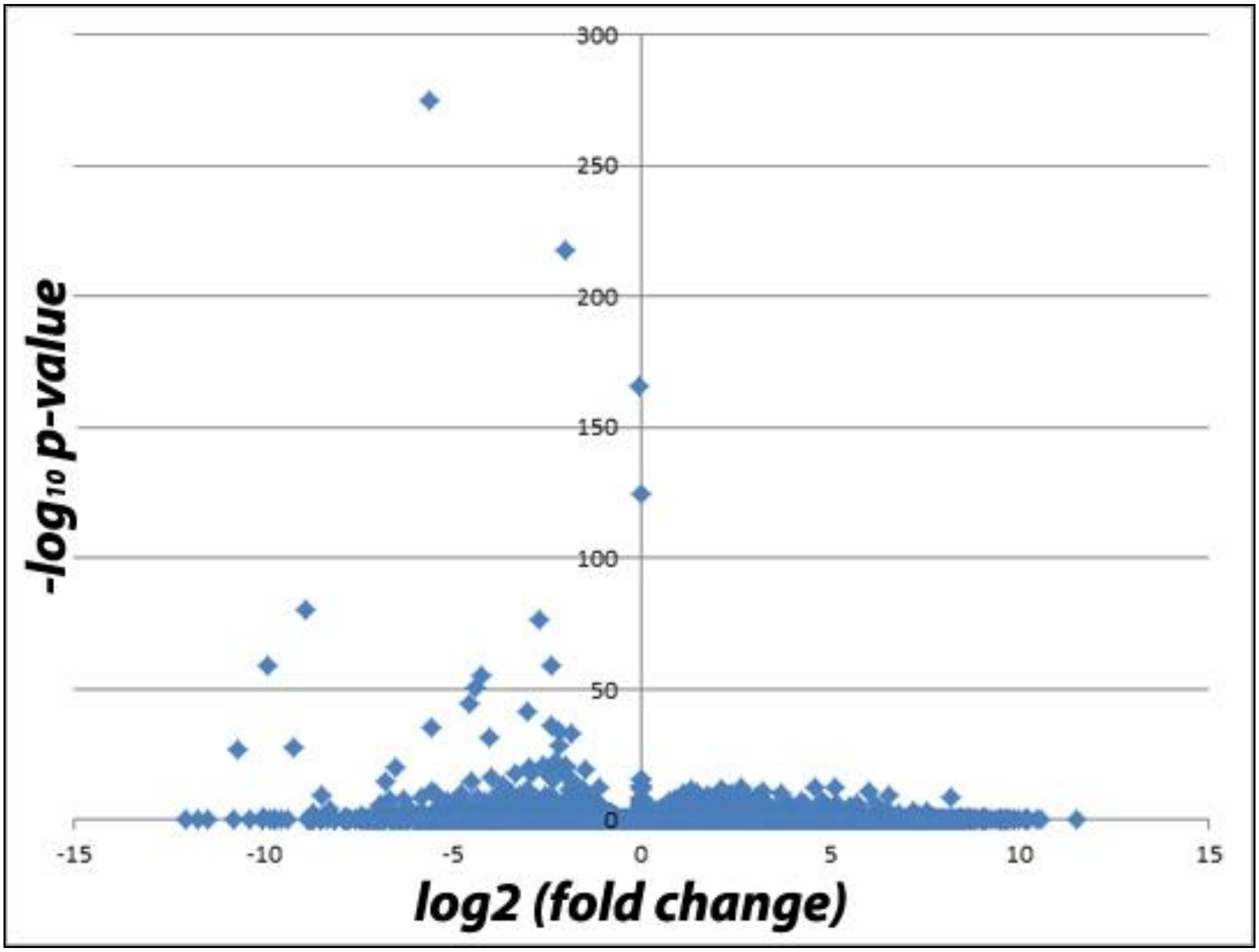

Supplemental Figure S3

Volcano plot showing the global transcriptional changes in FG clams as assayed by RNAseq. The log 2-fold change in the FG clams versus F1 is represented on the x-axis. The y-axis shows the $\log_{10}$ of the p value.
